# Enhanced hypocrellin production of *Shiraia* sp. SUPER-H168 by overexpression of alpha-amylase gene

**DOI:** 10.1371/journal.pone.0196519

**Published:** 2018-05-03

**Authors:** Ruijie Gao, Zhecun Xu, Huaxiang Deng, Zhengbing Guan, Xiangru Liao, Ye Zhao, Xiaohui Zheng, Yujie Cai

**Affiliations:** 1 Key Laboratory of Industrial Biotechnology, School of Biotechnology, Jiangnan University, Wuxi, Jiangsu, China; 2 College of Life Sciences, Northwest University, Xi’an, Shanxi, China; Universite Paris-Sud, FRANCE

## Abstract

Relative expression levels of twenty-four amylase genes in *Shiraia* sp. SUPER-H168 were investigated by real-time quantitative PCR when various carbohydrates, including glucose, sucrose, maltose, amylose, amylopectin and corn flour, were used as carbon source. Genes, including an α-glucosidase gene *Amy33* (2997 bp), an α-amylase gene *Amy365-1* (1749 bp) and a glycogen debranching enzyme gene *Amy130* (2487 bp), were overexpressed, and four overexpression transformants were constructed, respectively. When *Amy365-1* and *Amy130* were co-overexpressed, relative expression levels of seven hypocrellin biosynthesis genes and four related genes in central carbon catabolism were all increased. Expression of *Amy33* was also increased along with increase of *Amy365-1* and *Amy130*. Under liquid state fermentation, biomasses and hypocrellin productions were both gradually increased in four overexpression strains than those of wild type strain. Under SSF, hypocrellin production of *Amy365-1* and *Amy130* co-expression strain reached 71.85 mg/gds, which was 2.83 fold than that of wild type strain, and residual sugar was decreased from 35.47% to 16.68%. These results can provide a practical approach for other secondary metabolites by filamentous fungi under SSF when raw starch material is used as carbon source.

## Introduction

Photodynamic therapy is a minimally invasive therapeutic modality to treat with cancer diseases and many other non-malignant diseases [1,2]. As non-toxic dyes, photosensitizers (PSs) react with oxygen to generate reactive oxygen species (ROS) under illumine condition. These ROS can damage cellular constituents and cause cell death [3].

Hypocrellins, including hypocrellin A, hypocrellin B, hypocrellin C and hypocrellin D [4], are the important PSs of perylenequinone class. These compounds are isolated from the stromata of *Hypocrella bambusae* and *Shiraia bambusicola*. Hypocrellins and their derivatives [5–7] have gained considerable attentions for two decades, because they can accumulate selectively in cancer cells and their excellent light-induced antiviral activities against tumours and viruses, especially the antiviral activities against the human immunodeficiency virus (HIV) [8]. In addition to benefiting the pharmaceutical industry, hypocrellins also have extensively potential applications in the agricultural, cosmetic, food, and feed industries [9–11]. *S*. *bambusicola* is a parasitic fungus on twigs of several genera of bamboos. *S*. *bambusicola* was classified into the order of *Pleosporales* as AMANO [12] proposed and was recorded in Dictionary of the fungi [13], Cheng et al. [14] suggested that it might be positioned in the family of *Phaeosphaeriaceae*. So far, hypocrellins were mainly obtained from natural extraction, while the production was too low to meet its current medical demand.

In our previous work, we isolated a high-yield hypocrellin producing strain, *Shiraia* sp. SUPER-H168 [15], and hypocrellins produced by *Shiraia* sp. SUPER-H168 under solid state fermentation (SSF) have been identified. At the same time, corn was found to be the best substrate after evaluating eight kinds of agro-industrial crops and residues [16]. Compared with liquid state fermentation, SSF offers numerous advantages, including high volumetric productivity and relatively higher concentration of the products. SSF was found to be the best fermentation of industrialized production for hypocrellin production with *S*. *bambusicola*. Cracked corn is produced from maize endosperm in which starch is the major constituent. Normal corn starch consists of about 75% branched amylopectin and about 25% amylose, which is linear or slightly branched [17]. In previous studies, residual starch of dry fermented substrate remained at a high level (data was showed in this study). In order to increase the use ratio of corn starch, different amylases were added to solid fermented substrate, stimulating hypocrellin production. However, homogeneity of hypocrellin production of solid fermented substrate was not very good under the condition of amylase addition (data was not showed in this study). In order to improve the yield of hypocrellin and increase the use ratio of corn substrate, relative expression levels of twenty-four amylase genes in *Shiraia* sp. SUPER-H168 were investigated, when various carbohydrates, including glucose, sucrose, maltose, amylose, amylopectin and corn flour, were used as carbon sources. Furthermore, specific amylase genes were selected and overexpressed. In short, we want to evaluate the relationship between nutrient utilization (such as corn starch) and biosynthesis of hypocrellins.

When specific amylase genes were selected and then overexpressed, the relations between of relative expression levels of amylase genes and those of hypocrellin biosynthesis genes were also investigated. Related genes of hypocrellin biosynthesis, including FAD/FMN-containing dehydrogenase gene (*fad*), Salicylate 1-monooxygenase gene (*mono*), Zinc finger transcription factor gene (*zftf*), O-methyltransferase gene (*Omef*), major facilitator superfamily gene (*msf*), polyketide synthase gene (*pks*) and multicopper oxidase gene (*mco*), were reported by Deng et al [18].

Hypocrellin is a polyketide which is usually synthesized from acetyl-CoA and malonyl-CoA. In addition, hypocrellin is synthesized from one molecule of acetyl-CoA and six molecules of malonyl-CoA [18,19]. Pyruvate, the end-product of glycolysis, is oxidized to acetyl-CoA and CO_2_ by pyruvate dehydrogenase complex (PDH) [20]. Cytosolic acetyl-CoA is generated from pyruvate via acetaldehyde and acetate by the actions of pyruvate decarboxylase (PDC), acetaldehyde dehydrogenase (ALD) and acetyl-CoA synthetase (ACS) [21]. Acetyl-CoA is chemical precursor of malonyl-CoA in central carbon metabolism. Acetyl-CoA carboxylase (ACC) catalyzes acetyl-CoA to cytosolic malonyl-CoA. Suitable precursor provision is essential for the synthesis of hypocrellin. Therefore, relative expression levels of related genes in central carbon metabolism for efficient conversion of sugars into acetyl-CoA were also investigated.

**Fig 1:**
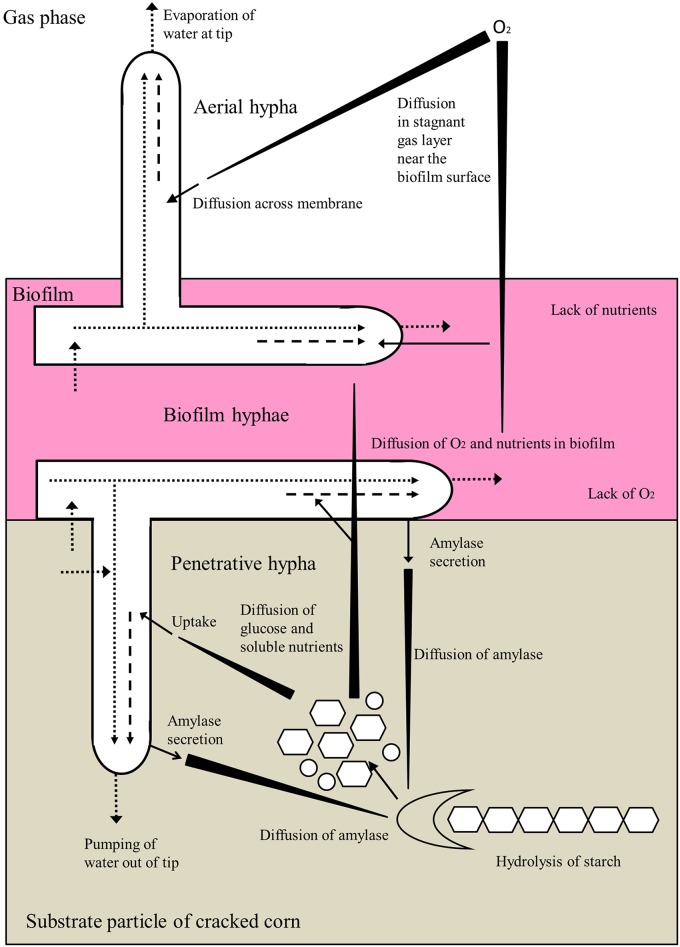
Phenomena involved in the growth of biofilm, penetrative, and aerial hyphae. “*Long triangles*” represent diffusion down concentration gradients. “*Dotted arrows*” represent flow of water or cytoplasm. “*Dashed arrows*” represent transport of vesicles.

**Fig 2:**
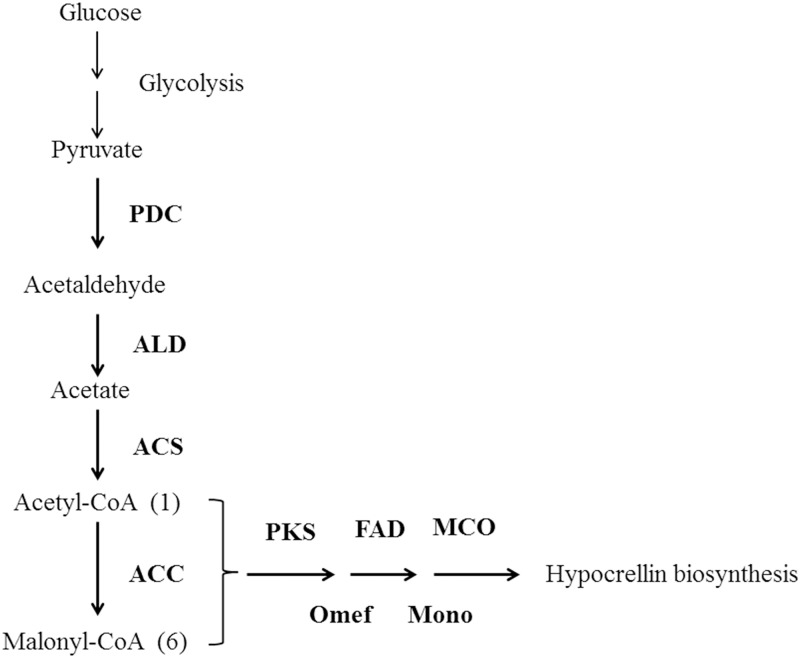
Schematic representation of hypocrellin biosynthesis pathway from glucose to hypocrellin.

In conclusion, cracked corn was used as solid substrate for SSF, specific amylase gene overexpression strains were constructed. Starch is the main constituent of cracked corn. Amylase was secreted by penetrative hypha of *S*. *bambusicola*, and then diffused to starch in substrate particle of cracked corn. Starch was hydrolyzed by amylase, and glucose or oligosaccharide was produced. Glucose was diffused and absorbed by hyphae of *S*. *bambusicola*. Fig 1 shows phenomena involved in the growth of biofilm, penetrative, and aerial hyphae [22]. In cell, glucose is converted to pyruvate through glycolysis, and then pyruvate is converted to acetyl-CoA and malonyl-CoA. Finally, hypocrellin was synthesized by hypocrellin biosynthesis enzymes (Fig 2).

## Materials and methods

### Materials

Corn was purchased from local supermarket (self-brand, Wuxi, Jiangsu, China).

### Microorganism and medium

*Shiraia* sp. SUPER-H168 (CCTCC M 207104) was a stock culture of the Laboratory of Biochemistry, School of Biotechnology, Jiangnan University, Wuxi, Jiangsu Province, China [15]. *Shiraia* sp. SUPER-H168 was routinely maintained on PDA slants at 4 °C by regular sub-cultivation (no longer than 3 months).

Seed culture medium (50 mL) which was potato dextrose medium was carried out in a Erlenmeyer flask (250 mL).

Liquid fermented medium was as follows: 5% carbohydrate with different carbon sources (glucose, sucrose, maltose, amylose, amylopectin and corn flour), 1% yeast extract, 228 mg/L K_2_HPO_4_, 174 mg/L K_2_SO_4_, 294 mg/L CaCl_2_, 492 mg/L MgSO_4_∙7H_2_O, 0.1 mg/L ZnSO_4_, 0.08 mg/L CuSO_4_ and 0.06% Tween 80.

Solid-state medium was carried out in a Erlenmeyer flask (250 mL) with 40 g cracked corn, and then moistened with salt solution which included glucose (1.65%, w/w, g/g dry substrate) and NaNO_3_ (0.43%, w/w) till 50% initial moisture contents.

### RNA isolation and first strand cDNA synthesis

RNA was isolated using a Takara RNA Extraction Kit (Takara, Ōtsu, Japan). To eliminate genomic DNA, RNA samples were treated with RNase-free DNase I (Takara). The quantity and quality of the total RNA extracted was determined using a NanoDrop-2000C spectrophotometer (Thermo Scientific, Wilmington, DE, USA) and the integrity was evaluated by analyzing the ratio between rRNA subunits of 18 S and 28 S after electrophoresis. The first strand cDNA was synthesized by reverse transcribing 500 ng RNA with 5×All-In-One RT MasterMix (Applied Biology Materials Inc., Richmond, Canada), and cDNA sample were stored at -20 °C [23].

### Real-time quantitative PCR (RT-qPCR)

*Shiraia* sp. slf14 has been genome sequenced (GenBank: KM434884.1). The homology between *Shiraia* sp. slf14 and SUPER-H168 was 99%. We downloaded the genome sequence of *Shiraia* sp. slf14, then the genes in this study were blasted with various amylase conserved domain in biolinux 7.0. According to the National Center for Biotechnology Information (NCBI), twenty-four amylase genes were selected for further study. Primers and glycoside hydrolase (GH) family of amylase genes were showed in Table 1. To standardize the relative quantification of the cDNA, glyceraldehyde-3-phosphare dehydrogenase (*gpd*) gene was selected as an endogenous control gene.

**Table 1:** Primers and relevant information of reference and target genes.

**Gene symbol**	**Gene name**	**GH**	**Primers (5′- 3′)**	**Amplification size (bp)**	**R**^**2**^	**RT-qPCR Efficiency**
*Amy1*	α-glucosidase	31	F: TCTGTGTCCTTGTATCTA	89	0.985	1.959
			R: TTGTAGTTGATGGTTGAA			
*Amy33*	α-glucosidase	31	F: AGTCACTCAATCTCAAGGTAG	79	0.992	1.976
			R: GAGGCGTCCAAGTATGTT			
*Amy68*	glucoamylase	15	F: ATTCTATCTTATCGTCTA	194	0.997	1.989
			R: ATATACATCTTCTGAGTA			
*Amy69*	Glycogen debranching enzyme	63	F: GACGACTGTTATCTGGTA	83	0.989	1.985
			R: CTGGTTCATCCTTATCCT			
*Amy179*	Glycogen debranching enzyme	13 or 133	F: ATTCCACACCTATTCATCTT	97	0.994	1.967
			R: TCAGAACCAGAGAACAAC			
*Amy257*	glucoamylase	15	F: CGTCAACAACAATTACTTCAG	90	0.996	1.987
			R: CGCAACATTAGCCAGTAG			
*Amy275*	α-glucosidase	31	F: ATACAACTTGGATACTACC	94	0.992	1.988
			R: AATGATGATGACCTTCTC			
*Amy279*	α-glucosidase	31	F: ACCAGTATCCAGAACCAT	79	0.987	1.963
			R: GCGAAGCAATAGACATCA			
*Amy289*	α-glucosidase	31	F: GAAGGCGAGAAGATTGAT	100	0.994	1.979
			R: CAGATGCTAAGTAGTTGAATG			
*Amy49*	α-amylase	13	F: AACCTTGTAGACGACCAT	86	0.996	1.998
			R: CAGCCATACTTCTCTTCAG			
*Amy82*	α-glucosidase	31	F: ATACACCATACAGTCTTCTTG	81	0.989	1.986
			R: AACTAACTCCGTCATCCA			
*Amy102*	α-amylase	13	F: GTCAGATAGCAAGCACTA	83	0.998	1.978
			R: GAGGAAGCCAGATGTTAT			
*Amy122*	α-amylase	13	F: AATTGGTGTTGATGTCAT	79	0.997	1.959
			R: GTAGTCTCTGATGTCGTA			
*Amy130*	Glycogen debranching enzyme	63	F: TTGAATATAACTGAGGATAA	192	0.992	1.989
			R: ATGTGAAGAATAGAGAAC			
*Amy207*	α-amylase	13	F: GCTCGCTATGTTCGTTGTC	75	0.987	1.976
			R: GCATTCACCATTCCAATCTCTT			
*Amy209*	α-glucosidase	31	F: GACAACAATCCATTCAAC	79	0.992	1.993
			R: AGCGTAGTGTATCTTCTA			
*Amy219*	α-amylase	13	F: ATTGACTATATCGCTTCT	175	0.979	1.994
			R: TCTTCATATTGTTCTTGTG			
*Amy229*	α-amylase	13	F: TGATGTTCCTGGTATTAC	120	0.996	1.985
			R: TAATCAGCAATGTCGTAT			
*Amy322*	α-glucosidase	31	F: TACAACGGTATCAAGGAA	123	0.993	1.993
			R: GTCATTGGTTACAGAAGG			
*Amy365-1*	α-amylase	13	F: TGGATTACGCTACTTATTAC	94	0.998	1.996
			R: GTATTGCTAACGGTATTCA			
*Amy365-2*	α-amylase	13	F: ATCGCAACCTGTTATCTC	76	0.991	1.987
			R: ATGGTCGCCGTTATTATC			
*Amy381*	glucoamylase	15	F: GAGAACTGATGGATGGAA	98	0.987	1.957
			R: ACATACATAGTCTGCGATT			
*Amy406*	α-glucosidase	31	F: CCTGCTGAATGTTATGGAA	79	0.988	1.968
			R: CAATATGCTAATGCTGAAGTC			
*Amy444*	α-glucosidase	31	F: TACCTCTTCTTGTTCGTA	84	0.978	1.984
			R: AATTGAGCCAGTCATAAC			
*gpd*	Glyceraldehyde-3-phosphate dehydrogenase	—	F: TTGACCTGACTGTCCGCATC	209	0.999	1.988
			R: CGAGACGAGCTTGACGAAGT			

The amplification efficiency (E) of all primers sets was tested with serial dilutions of template cDNA and calculated from the slope of the dilution curve according to the equation:

$$\text{E}=\left[10^{-(1/\text{slope})}-1\right]\times 100\%.$$

The expressions of the amylase genes were assessed in hyphae grown in liquid fermented media with different carbon sources. RT-qPCR reactions were carried out in 96-well block with a CFX96 Real-Time PCR Detection System (Bio-Rad, Hercules, CA, USA). The thermal profiles were performed using the following conditions: 95 °C 10 min, 30 cycles of 95 °C for 3 s, 57 °C for 30 s, 72 °C for 30 s. In order to evaluate the specificity of primer sets used for RT-qPCR amplification, the melting curve was analyzed. All amplifications were performed in triplicate.

Relative expression levels of amylase genes were calculated based on the threshold cycle (C_T_) deviation of the treated sample versus a control and expressed in comparison to reference gene. In this study, medium with glucose as carbon source was selected as a control.

$$\text{Relative}\;\text{expression}\;\text{ratio}=2^{\left[\Delta \text{CT}\left(\text{control}\right)-\Delta \text{CT}\left(\text{treated}\right)\right]}.$$

### Cloning of the full-length cDNA of the amylase genes and plasmid construction

**Fig 3:**
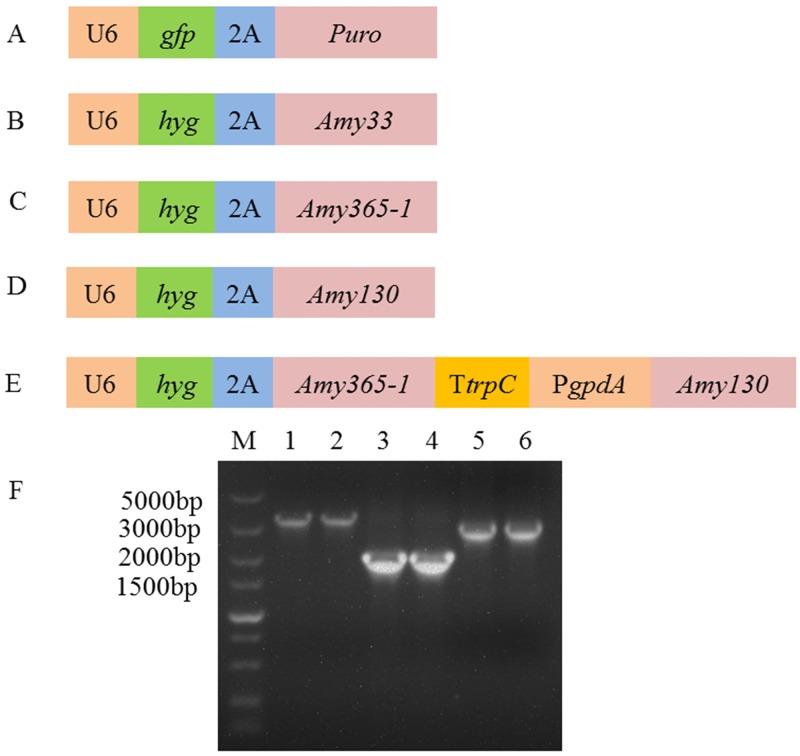
Constructions of expression vectors and cDNA of amylase gene in this study. (A) PgfpPuro, (B) PhygAmy33, (C) PhygAmy365-1, (D) PhygAmy130 and (E) PhygPgpdAAmy365-1-Amy130. U6, U6 promoter; P*gpdA*, gpdA promoter; T*trpC*, the *trpC* terminator; *hyg*, gene of protein resistant to hygromycin B; 2A, 2A peptide as a coexpression linker; *Amy33*, α-glucosidase gene; *Amy365-1*, α-amylase gene; *Amy130*, glycogen debranching enzyme gene. (F) cDNA of amylase gene. Lane M: DL5000 DNA Marker; lanes 1–2: cDNA of *Amy33*; lanes 3–4: cDNA of *Amy365-1*; lanes 5–6: cDNA of *Amy130*.

Deng et al. [18] has determined that a lentiviral expression vector, PgfpPuro, was chosen as an expression plasmid of *S*. *bambusicola*. The vector comprises U6 promoter, green fluorescent protein gene *gfp*, 2A peptide, and gene of the protein resistant to puromycin *Puro* (Fig 3A). On the basis of PgfpPuro, Phyg expression vector and PhygPgpdA co-expression vector were constructed in this study.

The Phyg expression vector was constructed by the following process. First, the gene *hyg* of Hyg protein was used to replace the *gfp* of the PgfpPuro expression plasmid. The PgfpPuro vector was linearized through amplification reaction with VF1 and VR1 primers. The Hyg protein gene *hyg* was amplified from pUCATPH plasmid with primers hygF and hygR. The amplified *hyg* with 15-bp nucleotides (the underlined sequences in Table 2) was reverse-complemented with the linearized lentiviral vector. On the basis of the 15-bp reverse-complement nucleotides between linearized lentiviral vector and amplified *hyg*, the ligation-free cloning kit (ABM Inc., Richmond, Canada) was used to construct Phyg vector by homologous recombination.

**Table 2:** Primers were used for construction of overexpression plasmids.

**Primer symbol**	**Primers (5′- 3′)**
VF1	GGTGGCACTAGAGAAGACTATTTTTG
VR1	GAATTCGGAAGCGGGCAGTG
hygF	TTCTCTAGTGCCACCATGAAAAAGCCTGAACTCACCG
hygR	CCCGCTTCCGAATTCTTCCTTTGCCCTCGGAC
VF2	GGATCCGGGGCCGGGGTTGCTC
VR2	ACGCGTTCCGGAAATCAACCTCTGG
Amy33F	CCCGGCCCCGGATCCATGGCGCGCTCAAGCT
Amy33R	ATTTCCGGAACGCGTCTACGTCCAACTCAAACTCCAC
Amy365-1F	CCCGGCCCCGGATCCATGTCGGCTCCTCGTCCACGAGGC
Amy365-1R	ATTTCCGGAACGCGTTTATTGCCATGTACTATTCACACTCGAAGCC
Amy130F	CCCGGCCCCGGATCCATGCGCCTTCCATCATC
Amy130R	ATTTCCGGAACGCGTCTACCTCCGTCTCCAACC
TtrpCF	ATCCACTTAACGTTACTGAAATCATC
TtrpCR	CATTTTTCGACAATCCGAGTGGAGATGTGGGAG
PgpdAF	GATTGTCGAAAAATGTAAAGG
PgpdAR	GATGAAGTGTTTGTTGAGCTAGGGGAG
Amy365-1R1	TAACGTTAAGTGGATTTATTGCCATGTACTATTCACACTCGAAGCC
Amy130F1	AACAAACACTTCATCATGCGCCTTCCATCATC

In order to overexpress *Amy33*, overexpression plasmid PhygAmy33 (Fig 3B) was constructed. The gene of Puro protein was replaced by *Amy33*. It was constructed in the same way as construction of Phyg vector with linearized plasmid which was amplified with primers VF2 and VR2 and *Amy33* which was amplified with primers Amy33F and Amy33R. PhygAmy365-1 (Fig 3C) and PhygAmy130 (Fig 3D) were also constructed in the same way as PhygAmy33. *Amy365-1* was amplified with primers Amy365-1F and Amy365-1R, and *Amy130* was amplified with primers Amy130F and Amy130R.

In order to co-overexpress *Amy365-1* and *Amy130*, co-overexpression plasmid PhygPgpdAAmy365-1-Amy130 (Fig 3E) was constructed. It was consisted of the following 5 fragments (in order): (1) linearized vector amplified from plasmid Phyg with primers VF2 and VR2; (2) *Amy365-1* was amplified with primers Amy365-1F and Amy365-1R1; (3) the T*trpC* terminator amplified from pAN52-1Not (GeneBank accession no. **Z32524**); (4) the P*gpdA* promoter was amplified from genomic DNA of *S*. *bambusicola* with primers PgpdAF and PgpdAR. P*gpdA* promoter had been identified (Deng et al. 2017) and chosen as the native promoter for overexpression in *S*. *bambusicola*. (5) *Amy130* was amplified with primers Amy130F1 and Amy130R. They were integrated by homologous recombination, and then PhygPgpdAAmy365-1- Amy130 was constructed.

Three amylase genes included *Amy33* (GenBank accession no. **MF535182**), *Amy365-1* (GenBank accession no. **MF535183**) and *Amy130* (GenBank accession no. **MF535184**) were cloned and deposited in GenBank. All primers used in this study and reverse-complement nucleotides with underlined were showed in Table 2.

### Preparation of protoplasts and fungal transformation

The procedures for fungal protoplast generation and transformation were carried out as described by Deng et al. [18]. Spore was selected as the original host and the cell wall was eliminated after 4 h degeneration with enzyme mixture. Overexpression plasmid (5μg) was added to 200 μL of 2–5×10^6^ protoplasts in STC solution (10 mM Tris-HCl and 10 mM CaCl_2_ at pH 7.5), and 100 μL of cold PTC buffer (50% PEG 3350, 10 mM Tris-HCl and 10 mM CaCl_2_ at pH 7.5) was then added to the mixture. After incubation on ice for 20 min, it was kept at room temperature for 15 min and then centrifugated. Protoplasts were suspended in regeneration medium and incubated with shaking at 75 rpm and 30 °C until the cell walls were recovered. Then protoplasts were spread onto the regeneration agar medium with 500 μg/mL of hygromycin B.

### Screening of transformants

Transformant selections were based on a two-step screening method. Hygromycin B resistance was used for the first round screening. Potential transformants were further cultivated on potato dextrose medium with 500 μg/mL hygromycin B. Plasmids in potential transformants were isolated using a Filamentous Fungal Plasmid DNAout Kit (Tiandz, Beijing, China) according to manuscript’s protocol. PCR was performed using plasmid as template DNA, and then the plasmids were sequenced by Sangon Biotech.

### Amylase overexpression and analysis of hypocrellin production

Corn was the best carbon source for hypocrellin production under SSF. In order to improve the utilization of this carbon source, four amylase overexpression transformants were cultured using corn flour as carbon source. Wild type *Shiraia* sp. SUPER-H168 was chosen as the control strain to analyze different parameters, including biomass, hypocrellin production, amylase activity, residual sugar and relative expression levels. RNA was isolated from five strains at 48, 72 and 96 h. The relative gene expression levels were studied. Therein, hypocrellin biosynthesis genes contained *fad*, *mono*, *zftf*, *Omef*, *msf*, *pks*, and *mco*. Related genes in central carbon metabolism comprised *pdc*, *ald*, *acs*, and *acc*. The primers used for RT-qPCR analysis of hypocrellin biosynthese genes and related genes were showed in Table 3. Hypocrellin was produced, extracted and detected as described by Cai et al. [24].

**Table 3:** Primers of hypocrellin biosynthesis genes and related genes in central carbon metabolism.

**Gene symbol**	**Gene name**	**Primers (5′- 3′)**
*fad*	FAD/FMN-containing dehydrogenase	F: TGTGACCGCCATCACCTTAC
		R: TTGTCGTATGGGTGGGAAGC
*mono*	Salicylate 1-monooxygenase	F: TCTCGGGGAATTATGGCACG
		R: ACAACCGTTCTCGCATCAGT
*zftf*	Zinc finger transcription factor	F: GAACACCGTCGCAAGATTCG
		R: TCATTGGCATCGCTTGGAGT
*Omef*	O-methyltransferase	F: GAACTACCTGAAGGCACGCT
		R: GCTCGGAAGGATACTCGCTC
*msf*	Major Facilitator Superfamily	F: TCCCGTAGCCTTGCTTTCTG
		R: CCGGCTTCTTCTTGACGCTA
*pks*	Polyketide synthase	F: TGCTGAGGTAGCAGTCAAGC
		R: TTATGCTACGGTCGTCGCTC
*mco*	Multicopper oxidase	F: TATGGCGCTACGAGTGGAC
		R: ACTCCCTGGCCGATAACGTA
*pdc*	pyruvate decarboxylase	F: ATTGTAACGAACTGAATGCT
		R: GTGGTGACTATGGCTGAA
*ald*	acetaldehyde dehydrogenase	F: GTTGGCAGTGAGAATGGA
		R: CTGTTGCGTAGTTGATGATG
*acs*	acetyl-CoA synthetase	F: GTTGGCTTATACGCTCAA
		R: TTCTGGAATCATAGGTAGGT
*acc*	acetyl-CoA carboxylase	F: ATCTCAACTGCCGAATACA
		R: AGTGCCAACAATCTCCAA

### Solid state fermentation

Fermentation was carried out at 30 °C for 15 days under relatively humidity high than 95%. The optimized SSF conditions were as follows: substrate particle size 0.8–1 mm, initial moisture content 50%, and temperature 30 °C. The optimum compositions of the supplementary glucose and NaNO_3_ were 1.65% and 0.43% (w/w), respectively [16]. The samples were removed at regular intervals of 48 h after agitation and were analyzed for different parameters. Fermented substrate was dried at 60 °C and pulverized by passing through a 0.2 mm sieve. One gram sample was refluxed with 50 mL methanol in a water bath at 70 °C for 2 h. The extract was filtered through 0.45 μm membrane filter and diluted to 100 mL. After that, hypocrellins was determined by reverse-phase high-performance liquid chromatography (HPLC) [16].

### Alpha-amylase activity assay

For enzyme extraction, fermented product was ground in an ice-cold mortar, and then 10 mL of phosphate buffer (pH 7.0, 20 mM) per gram of fermented product was added and agitated for 4 h at 4 °C. Subsequently, the material was filtered and centrifuged (10,000 × *g* for 10 min) at 4 °C, and the supernatant (the enzyme extract) was used as the enzyme extract.

Alpha-amylase activity was assayed using 3,5-dinitrosalicylic acid (DNS) reagent according to Bernfeld [25]. The reaction mixture consisted of 0.5 mL of 1.0% (*w/V*) native corn starch (Product number S4126, amylose content of 27%, Sigma-Aldrich, USA), 0.4 mL of 0.1 M acetate buffer (pH 5.0), and 0.1 mL of appropriately diluted enzyme extract. After 30 min of incubation at 40 °C, the released reducing sugars (glucose equivalents) were measured by recording absorbance at 540 nm. Glucose was used as a standard. One unit (U) of α-amylase activity as defined as the amount of enzyme required to produce 1.0 μmol of glucose equivalent per min under under the conditions described above. The specific amylase activity, U/gds (grams of dry substrate), was calculated as the amylase units per gram of dry substrate in SSF.

### Biomass estimation

Total fungal biomass was estimated by measuring the N-acetyl glucosamine released by the acid hydrolysis of the chitin, present in the cell walls of fungi [26]. In brief, 0.2 g of dry fermented solid substrate was mixed with 1 mL of concentrated H_2_SO_4_ for acid hydrolysis. Acetyl acetone reagent (1 mL) was added to the mixture, which was then placed in a boiling water bath for 20 min. After cooling, ethanol (6 mL) was added, followed by the addition of 1 mL of Ehrlich reagent and incubation at 65 °C for 10 min. After cooling to room temperature, OD was measured at 530 nm against the reagent blank. N-acetyl glucosamine (Sigma-Aldrich) was used as the external standard.

### Residual sugar analysis

Residual sugar analysis was performed by the amyloglucosidase/α-amylase method (AOAC method 996.11) with the total starch assay kit from Megazyme (Wicklow, Ireland). Under SSF, cracked corn and solid substrates were dried at 80 °C for 24 h before analysis [27]. The residual sugar was also detected under liquid state fermentation.

### Statistical analysis

All the cultures and assays were replicated in triplicates and the results are mean standard deviations (±SD) of the values.

## Results

### RNA validation

RNA samples isolated from various culture conditions with different carbon sources were assessed for integrity and quality. All absorbance ratios at 260/280 nm of RNA samples ranged from 1.8 to 2.0. Agarose gel electrophoresis revealed no degradation. RT-qPCR included twenty-four amylase genes and a glyceraldehyde-3-phosphare dehydrogenase (*gpd*).

### Comparison of the expression levels of amylase genes

**Fig 4:**
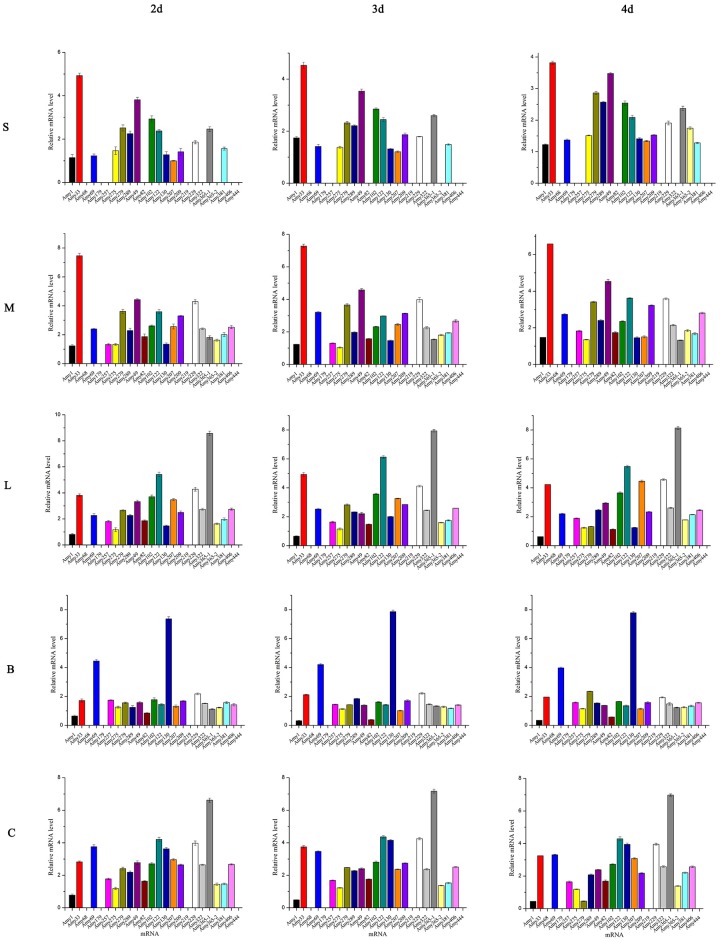
Relative mRNA levels of twenty-four genes when *Shiraia* sp. SUPER-H168 was cultured on different carbohydrates. Glucose was selected as a control and *gpd* was used as the reference gene for data normalization of relative expression level analysis. S, sucrose; M, maltose; L, amylose; B, amylopectin; C, corn flour.

Amylase gene expressions in *Shiraia* sp. SUPER-H168 were studied when various carbohydrates (glucose, sucrose, maltose, amylose, amylopectin and corn flour) were used as carbon sources (Fig 4). Four amylase genes, including *Amy68*, *Amy179*, *Amy219* and *Amy444*, were not expressed in all conditions with different carbohydrates. When sucrose was used as carbon source, five amylase genes, including *Amy257*, *Amy82*, *Amy322*, *Amy365-2* and *Amy406*, were not expressed (Fig 4S). When sucrose or maltose was used as carbon source, *Amy33* presented high expression (Fig 4S and 4M). When amylose was used as carbon source, *Amy365-1* had the highest expressive level (Fig 4L). When amylopectin was used as carbon source, Amy130 had the highest expressive level (Fig 4B). When corn flour was used as carbon source, six amylase genes including *Amy33*, *Amy69*, *Amy122*, *Amy130*, *Amy229* and *Amy365-1* had high expressive level (Fig 4C).

### Overexpression of amylases and screening of transformants

In order to improve the utilization of corn, we selected an α-glucosidase gene *Amy33*, an α-amylase gene *Amy365-1* and a glycogen debranching enzyme gene *Amy130* for further study. The length of *Amy33*, *Amy365-1* and *Amy130* were 2997 bp, 1749 bp and 2487 bp, respectively (Fig 3F).

**Fig 5:**
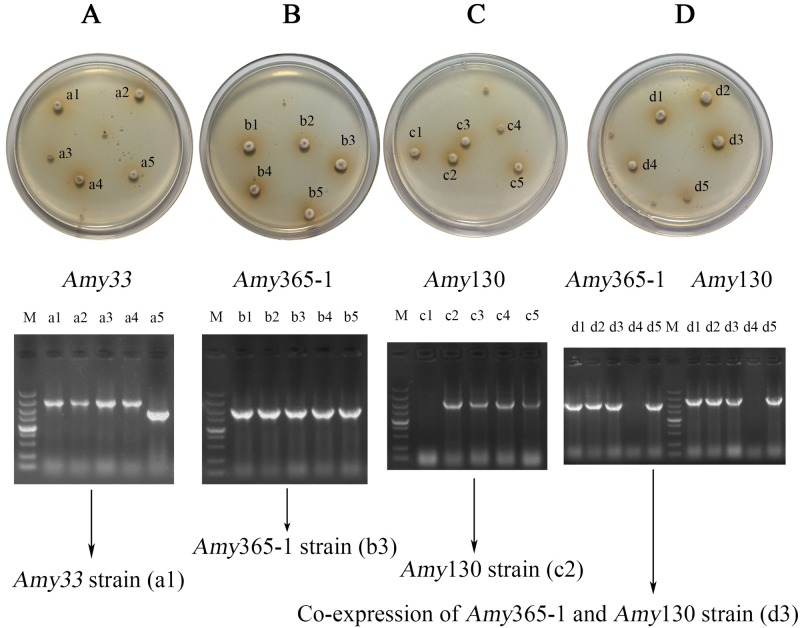
Screening of fungal transformants. *Amy*33 strain (A), *Amy*365-1 strain (B), *Amy*130 strain (C) and co-expression of *Amy*365-1 and *Amy*130 strain (D).

Four overexpression vectors were constructed, and then they were transformed into protoplasts. In order to efficiently screen candidates, a two-step screening method was used. After growing 3 days on PDA plates with 500 μg/mL hygromycin B (Fig 5). Potential transformants (a1-5,b1-5,c1-5 and d1-5) were further cultivated on potato dextrose medium with 500 μg/mL hygromycin B. Plasmids of candidates were isolated and PCRs were performed with different plasmids used as templates. Furthermore, plasmids were sequenced and one of targeted transformants (a1, b3, c2 and d3) were selected. Four amylase overexpressed transformants were *Amy*33 strain (a1), *Amy*365-1 strain (b3), *Amy*130 strain (c2) and co-expression of *Amy*365-1 and *Amy*130 strain (d3), respectively.

### Liquid state fermentation

Four overexpressed transformants were cultured using corn flour as carbon source. Wild type *Shiraia* sp. SUPER-H168 was chosen as the control strain for analysis of biomass, hypocrellin production, residual sugar and relative expression levels.

**Fig 6:**
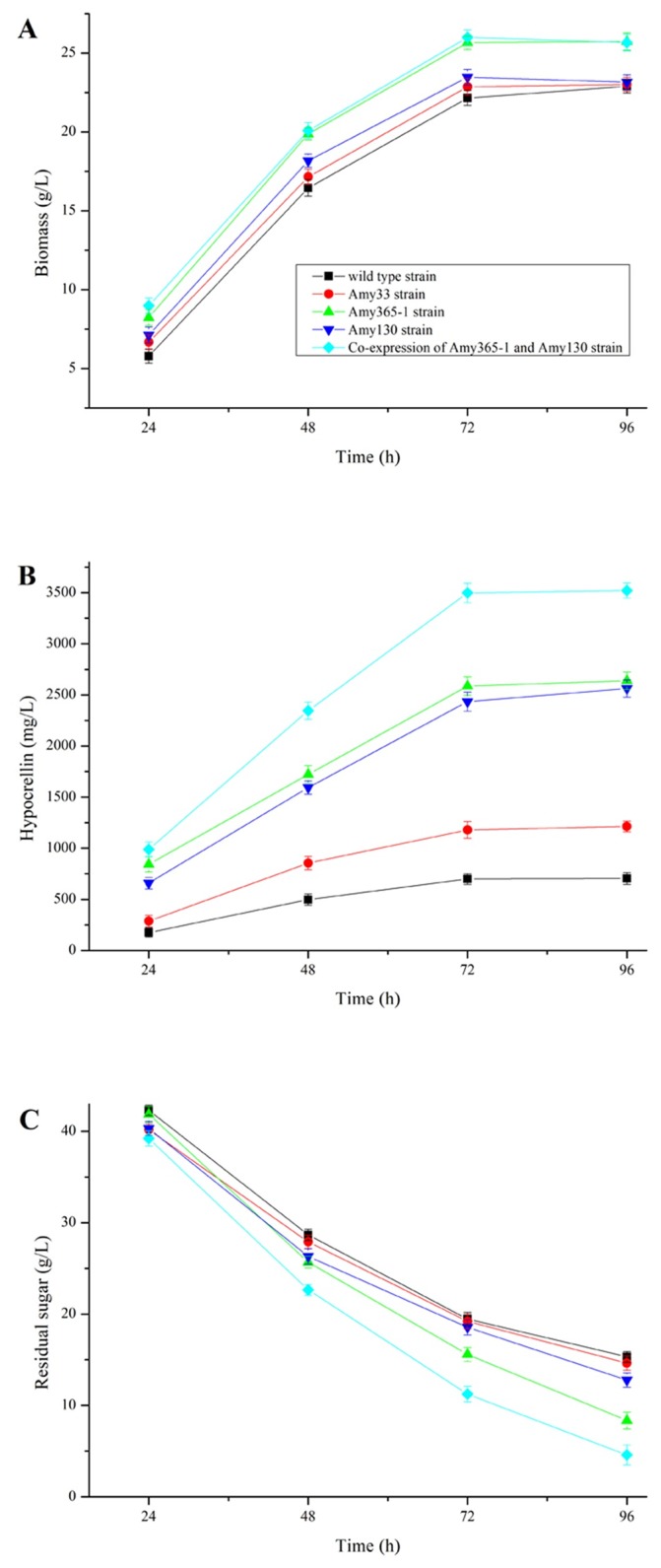
Biomasses (A), hypocrellin productions (B) and residual sugars (C) when five strains were cultured under liquid state fermentation.

Biomass (Fig 6A) and hypocrellin production (Fig 6B) were both gradually increased in five strains, at the same time, residual sugar was gradually decreased (Fig 6C). Four overexpression transformants all had positive effects on biomasses and hypocrellin productions at different culture times. In addition, hypocrellin production in co-expression strain of AMY365-1 and AMY130 reached the highest level of 3521 mg/L at 96 h, it was increased by about five times than wild type strain (703 mg/L at 96 h).

### RT-qPCR analysis of hypocrellin biosynthesis genes and related genes in central carbon metabolism

**Fig 7:**
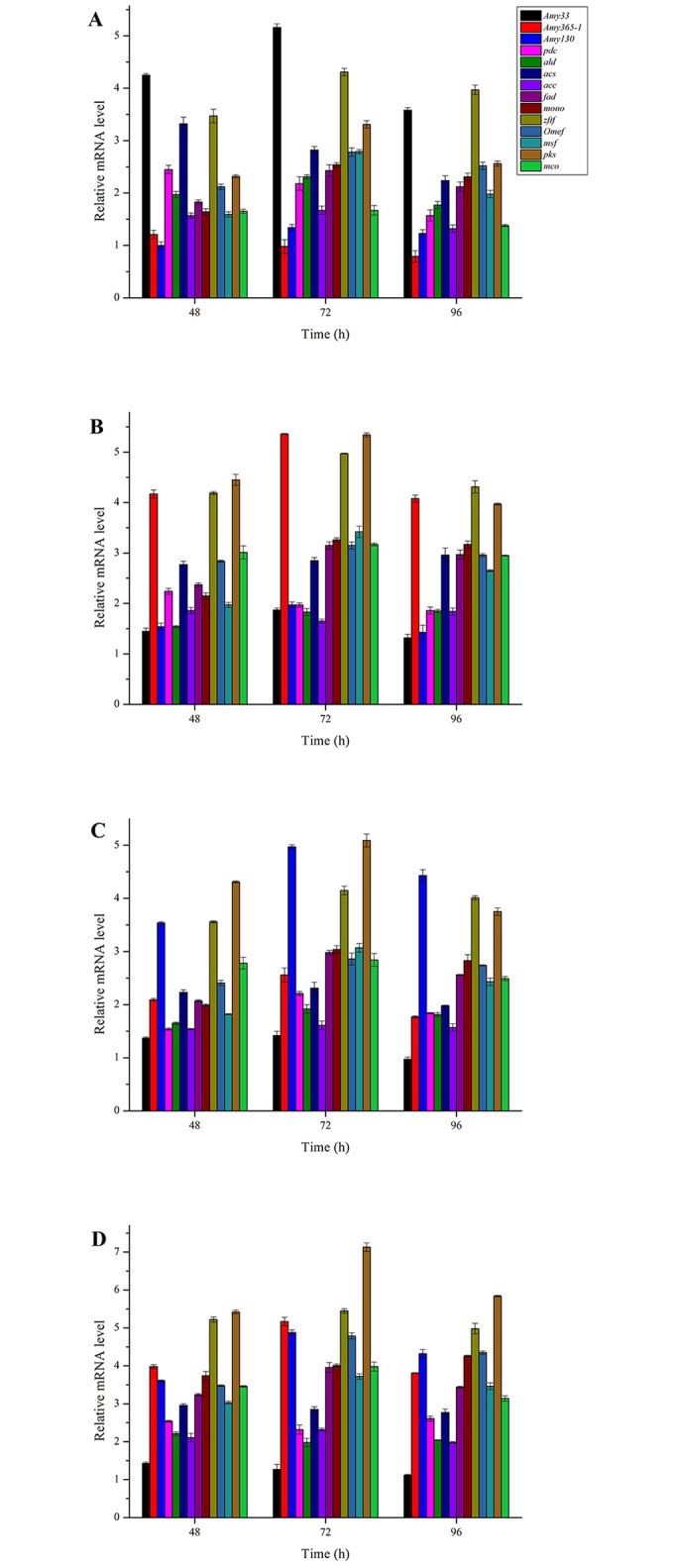
Relative mRNA levels of different genes when four overexpression strains were cultured on corn flour. (A) PhygAmy33, (B) PhygAmy365-1, (C) PhygAmy130 and (D) PhygPgpdAAmy365-1-Amy130.

In order to analyze the effects of amylase overexpression on genes of hypocrellin biosynthesis, relative expression levels of three amylase genes, seven hypocrellin biosynthesis genes and four related genes in central carbon metabolism were studied at 48 h, 72 h and 96 h (Fig 7). The relative expression levels of all these genes were increased in four overexpression strains than those of the control strain. These results suggested that there was a positive correlation among amylase genes, hypocrellin biosynthesis genes and related genes in central carbon metabolism. Relative expression levels of seven hypocrellin biosynthesis genes were all increased when AMY33, AMY365-1 and AMY130 were overexpressed, especially *zftf* and *pks* had an obvious increase in four overexpressed strains. Relative expression level of *Amy33* was increased when AMY365-1 or AMY130 was overexpressed (Fig 7B, 7C and 7D). *acs* also had an obvious increase compared with other *pdh* and *acc* in four overexpressed strains. In addition, hypocrellin biosynthesis genes reached their highest relative expression levels in PhygPgpdAAmy365-1-Amy130 constructed strain. The result agreed with hypocrellin production in liquid state fermentation.

### Solid state fermentation

**Fig 8:**
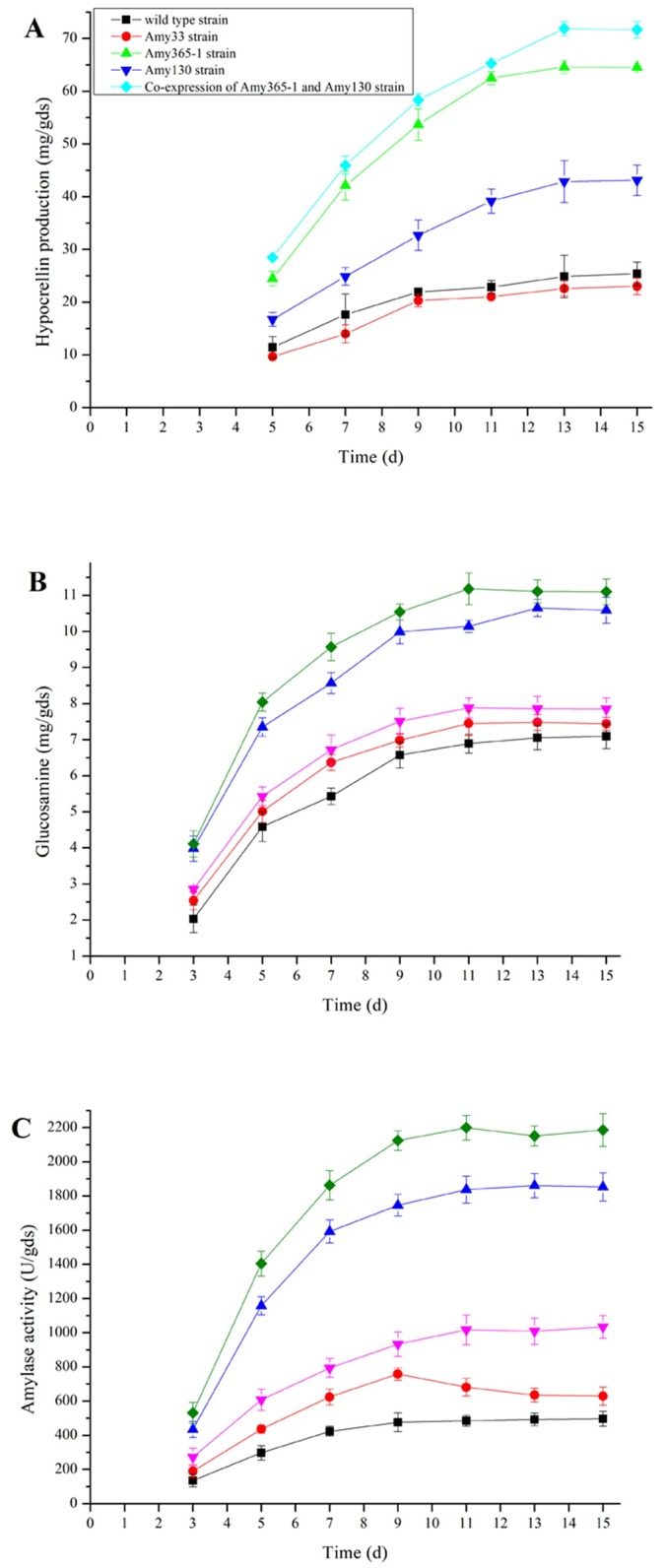
Hypocrellin productions (A), biomasses (B) and amylase activities (C) when five strains were cultured under SSF.

Hypocrellin productions, fungal biomasses and amylase activities under SSF were also studied in five strains (Fig 8).

Under SSF, hypocrellin production was increased in Amy365-1 or Amy130 overexpression strain, while the value was decreased in Amy33 overexpression strain compared with that of wild type strain (Fig 8A). In PhygPgpdAAmy365-1-Amy130 constructed strain, hypocrellin production reached 71.85 mg/gds within 13 d which was the highest yield in all studies, and it was 2.83 fold compared with wild type strain (25.37 mg/gds). Hypocrellin productions in PhygAmy33 constructed strain, PhygAmy365-1 constructed strain and PhygAmy130 constructed strain reached 23.01 mg/gds, 64.52 mg/gds and 43.11 mg/gds, respectively. Residual sugar of solid substrates at 15 d by PhygPgpdAAmy365-1-Amy130 constructed strain was 16.68%, compared with that of wild type strain was 35.47%.

Fungal biomasses (Fig 8B) and amylase activities (Fig 8C) were all increased in four overexpression strains compared with that of wild type strain. In PhygPgpdAAmy365-1-Amy130 constructed strain, glucosamine in fungal biomass reached 11.18 mg/gds within 11 d. Glucosamine in fungal biomass of wild type strain reached 7.09 mg/gds within 15 d. Amylase activity reached 2199 U/gds in PhygPgpdAAmy365-1-Amy130 constructed strain within 11 d which was the highest level in this study, and amylase activity reached 496 U/gds in wild type strain within 15 d. Amylase activity in Amy33 overexpression strain was increased in the first 9 d and was decreased from 9 d to 15 d.

## Discussion

In order to improve the yield of hypocrellins, a lot of efforts were made [28]. High-efficiency biosynthesis of hypocrellin A in *Shiraia* sp. using gamma-ray mutagenesis was investigated by Liu et al. Liu et al. [29]. In our previous studies, a high-yield hypocrellin producing strain *Shiraia* sp. SUPER-H168 was isolated [15]. At the same time, corn was found to be the best substrate after evaluating eight kinds of agro-industrial crops and residues [16]. Starch is the main constituent in corn, and it is also the main carbon source when hypocrellin is produced under SSF with *S*. *bambusicola*. In order to produce hypocrellin at an industrial scale in the future, the use ratio of corn substrate and the yield of hypocrellin are two key aspects under SSF, we need to select specific amylase for overexpression in *Shiraia* sp. SUPER-H168. In order to improve the yield of hypocrellin and increase the use ratio of corn substrate, relative expression levels of twenty-four amylase genes in *Shiraia* sp. SUPER-H168 using various carbohydrates, including glucose, sucrose, maltose, amylose, amylopectin and corn flour, as carbon sources were investigated, and specified amylases was selected to be overexpressed. Carbon source had important effects on development of microbiology and production of secondary metabolites. For example, fumonisin B1 production was induced by amylopectin in *Fusarium verticillioides* during colonization of maize kernels [27]. Results showed that *Shiraia* sp. SUPER-H168 contained luxuriant amylases, and twenty amylase genes were expressed when corn was used as carbon source. The other four amylase genes, including *Amy68*, *Amy179*, *Amy219* and *Amy444*, were not expressed when starchy material was used as carbon source, while corresponding reason for this issue was not clear. They may be expressed or induced by other polysaccharides.

An α-glucosidase gene *Amy33*, an α-amylase gene *Amy365-1* and a glycogen debranching enzyme gene *Amy130* had high relative expression levels when they were cultured on maltose, amylose and amylopectin, respectively. So they were selected to be overexpressed in *Shiraia* sp. SUPER-H168, and four transformants included PhygAmy33 strain, PhygAmy365-1 strain, PhygAmy130 strain and PhygPgpdAAmy365-1-Amy130 strain were constructed. The results were consistent with previous studies which reported that α-amylase was an inducible enzyme and was generally induced in the presence of starch or its hydrolytic product, maltose [30,31].

In recent years, a global interest has been focused on the raw-starch hydrolyzing amylases to simplify the procedure of starch digestion [32,33]. Cracked corn was used as substrate for SSF in this study and residual sugar of fermented substrate at 15 d by PhygPgpdAAmy365-1-Amy130 constructed strain was 16.68%. The result reflected that raw corn starch could be effectively hydrolyzed by amylases from *Shiraia* sp. SUPER-H168. Raw starch hydrolyzing α-amylase from *Bacillus licheniformis* under SSF by utilizing agricultural wastes was investigated [34]. The rate of hydrolysis of starch granules strongly depends on the botanical source from which they originated [35]. Lacerda et al. found that hydrolysis was more pronounced in the amorphous part of the starch granules by fungal α-amylase from *Aspergillus oryzae* [36]. This study also showed potentials of α-amylases from *Shiraia* sp. SUPER-H168 on raw starch hydrolyzing.

Hypocrellins produced by four overexpression strains were all increased under liquid state fermentation when corn was used as carbon source. While under SSF, when corn was used as carbon source, hypocrellin of PhygAmy33 constructed strain was decreased and biomass was increased compared with wild type strain. AMY33 which was an α-glucosidase was purified and studied in previous study, and it had a good glycoside hydrolysis with maltose [37]. In PhygAmy33 constructed strain, amylase activity was increased in the first 9 days and then was decreased. This might be due to the quantity of maltose or other starch hydrolytic product which acts as inducer was decreased after 9 d. The relative expression level of *Amy33* gene in genome of *Shiraia* sp. SUPER-H168 was reduced. When *Amy365-1* or *Amy130* was overexpressed, the relative expression level of *Amy33* was increased, this may be due to that more non-reducing ends of polysaccharides were present and α-glucosidase gene was induced [38]. The results reflected that α-glucosidase activity of *Shiraia* sp. SUPER-H168 under SSF was not a rate-limiting step. Furthermore, the main bottlenecks of amylase activities under SSF were the relative low expression levels of α-amylase and glycogen debranching enzyme. As a result, co-expression experiment of *Amy33* was not studied further.

In addition, relative expression levels of hypocrellin biosynthesis genes were increased along with overexpression of amylase gene. Presumably, hypocrellin production depended on starch catabolism, which accelerated carbon metabolism and ensured the timely and effective supply of glucose in cell. Glycolysis is a central pathway for the catabolism of carbohydrates which convers glucose to two pyruvate molecules [39]. And the pathway from pyruvate to acetyl-CoA and malonyl-CoA was enhanced, because relative expression levels of *pdc*, *ald*, *acs* and *acc* were increased when *Amy365-1* or *Amy130* was overexpressed. With respect to hypocrellin biosynthesis genes, *zftf* and *pks* were both obviously increased when amylase gene was overexpressed. ZFTF is a global transcription factor that mediates gene clusters of secondary metabolites [40]. Hypocrellin was increased along with the increase of relative expression level of *zftf* when amylase gene was overexpressed. PKS [18] is a key enzyme that catalyzed hypocrellin biosynthesis via repetitive decarboxylative claisen condensation of acetyl-CoA and malonyl-CoA. The relative expression level of *pks* was also increased along with the increase of that of *zftf*, and this was the main reason why hypocrellin increased.

Under SSF, using corn starch as carbon source can effectively reduce carbon metabolite repression [41]. The uptake and subsequent utilization of sugars derived from corn starch was conducive for hypocrellin biosynthesis. Woloshuk et al. also reported that the presence of glucose, maltose and maltotriose from corn starch, which was hydrolyzed by α-amylase from *Aspergillus flavus*, had a great influence on aflatoxin production [41]. After SSF, residual sugar of co-expression of *Amy*365-1 and *Amy*130 strain was 16.68%. While residual sugar of babassu cake under SSF by *Aspergillus awamori* IOC-3914 with mesophilic fungal amylases was 48% [42].

At the present time, many aspects of the interaction remain highly speculative, especially the events at the plasma membrane interface responsible for the sensing and uptake of sugars [43,44]. Furthermore, the components of the signal transduction network that mediate the interaction between the corn starch and the regulation of hypocrellin biosynthesis remain poorly understood.

In conclusion, *Shiraia* sp. SUPER-H168 had luxuriant amylases, and twenty amylase genes were expressed when corn was used as carbon source. When specific amylase gene was overexpressed, the relative expression levels of hypocrellin synthesis genes and related genes in central carbon metabolism were all increased. Under SSF, residual sugar was decreased from 35.47% to 16.68%, and hypocrellin production increased to 2.83 fold compared with that of wild type strain. At the same time, homogeneity of hypocrellin production of solid fermented substrate was good. This may be due to that amylases were mainly secreted by penetrative hyphae of *S*. *bambusicola*. Penetrative hyphae penetrate into cracked corn and secreted amylases in substrate particle. These results all lay the foundation for hypocrellin production at an industrial scale in the future, and they can provide a practical approach for other secondary metabolites by filamentous fungi under SSF when starch raw material is used as carbon source.
